# The increased analgesic efficacy of cold therapy after an unsuccessful analgesic experience is associated with inferior parietal lobule activation

**DOI:** 10.1038/s41598-022-18181-0

**Published:** 2022-08-29

**Authors:** Jae Chan Choi, Hae-Jeong Park, Jeong A. Park, Dae Ryong Kang, Young-Seok Choi, SoHyun Choi, Hong Gyu Lee, Jun-Ho Choi, In-Ho Choi, Min Woo Yoon, Jong-Min Lee, Jinhee Kim

**Affiliations:** 1 Department of Anesthesiology and Pain Medicine, Yonsei University Wonju College of Medicine, Wonju, 26426 Republic of Korea; 2 Cham Brain Health Institute, 08807 Seoul, Republic of Korea; 3 Department of Nuclear Medicine, Graduate School of Medical Science, BrainKorea21Project, Yonsei University College of Medicine, Seoul, 3722 Republic of Korea; 4 Alzza Health Institute, Seoul, Republic of Korea; 5 Department of Precision Medicine & Biostatistics, Yonsei University Wonju College of Medicine, Wonju-si, 26426 Republic of Korea; 6 Department of Electronics and Communications Engineering, Kwangwoon University, Seoul, 01897 Republic of Korea; 7 Department of Radiology, Yonsei University Wonju College of Medicine, Wonju-si, 26426 Republic of Korea; 8 Department of Practical Arts Education, Chinju National University of Education, Jinju-si, 52673 Republic of Korea; 9 Department of Architectural Design, Kaywon University of Art and Design, Uiwang-si, 16038 Republic of Korea; 10 Department of Biomedical Engineering, Hanyang University, Seoul, 4763 Republic of Korea; 11 School of Psychology, Korea University, Seoul, 2841 Republic of Korea

**Keywords:** Pain, Human behaviour

## Abstract

Prior experiences of successful and failed treatments are known to influence the efficacy of a newly applied treatment. However, whether that carry-over effect applies to non-pharmacological treatments is unknown. This study investigated how a failed treatment history with placebo analgesic cream affected the therapeutic outcomes of cold-pack treatment. The neural correlates underlying those effects were also explored using functional magnetic resonance imaging. The effect of the placebo analgesic cream was induced using placebo conditioning with small (44.5 °C to 43.7 °C, negative experience) and large (44.5 °C to 40.0 °C, positive experience) thermal stimuli changes. After the placebo conditioning, brain responses and self-reported evaluations of the effect of subsequent treatment with a cold-pack were contrasted between the two groups. The negative experience group reported less pain and lower anxiety scores in the cold-pack condition than the positive experience group and exhibited significantly greater activation in the right inferior parietal lobule (IPL), which is known to be involved in pain relief. These findings suggest that an unsatisfying experience with an initial pain-relief treatment could increase the expectations for the complementary treatment outcome and improve the analgesic effect of the subsequent treatment. The IPL could be associated with this expectation-induced pain relief process.

## Introduction

Placebo analgesia describes pain reduction associated with the belief or expectation that a treatment will relieve pain, even when the treatment itself is inert^1^. The placebo response is driven by both opioid-mediated mechanisms and non-opioid-mediated mechanisms, in which expectation plays a fundamental role^2–5^. Previous brain imaging studies have shown that placebo analgesia is accompanied by a decreased activity in brain regions related to pain and an increased activity in brain regions related to cognitive function^3,6,7^. Specifically, placebos have been found to reduce the activity of brain regions involved in the processing of sensory and affective aspects of nociceptive pain (e.g., somatosensory cortex, insula, thalamus, and anterior cingulate cortex). Activation of the dorsolateral prefrontal cortex and intraparietal sulcus increased during pain processing when pain relief was expected, indicating the construction of top-down representations of psychological contexts such as beliefs and expectations^7^.

Treatment history (prior experiences of success or failure with treatment) shapes patient’s expectations about treatment, and those expectations affect treatment outcomes^8^. Several studies have shown carry-over of a placebo analgesic effect to subsequent treatments that differ in pharmacological profile [patch or ointment]^9,10^ or administration routes [topical or oral]^11^, which could be explained by the learning principle of generalization^12,13^. Specifically, an unsuccessful/successful experience with a prior treatment decreased/increased the placebo analgesic effect of subsequent treatment^9–11^. In contrast, a study of chronic pain patients, those with a more unsuccessful treatment history exhibited larger placebo responses than those with a more successful treatment history^14^. In line with these findings, Zunhammer et al.^11^ found that the negative experience group reported increased expectations for the novel treatment, whereas the positive experience group showed decreased expectations suggesting that changing the treatment characteristics (e.g., route of drug administration from patch to pill) produced positive expectation for the new treatment but did not reduce the carry-over effect of negative treatment history. As all the treatments used in previous studies^9–11^ were pharmacological, they might not have differed enough to reduce this carry-over effect. Because of the similarities among treatments, the placebo effect might be generalized across treatments via associative learning^13^.

The current study utilized distinct interventions of pain relief (e.g., pharmacological cream and cold therapy) to explore the effect of expectations for the subsequent treatment based on the efficacy of the prior treatment. Cold therapy (e.g., cold-pack application) was chosen as an alternative non-pharmacological treatment after administering an inert analgesic cream for relief from the pain caused by noxious thermal stimulation. Cold-pack application is a widely used, classical modality of pain relief that is practiced in a broad range of medical areas and induces analgesic effects by decreasing perceived intensity of hot cutaneous stimuli and increasing pain thresholds^15^. The two treatments used in this study differ in terms of both administration (e.g., cold-pack and ointment) and type (e.g., pharmacological and non-pharmacological) to minimize the carry-over expectation for treatment efficacy caused by similar treatments^13,16^.

A placebo conditioning procedure was used to manipulate the prior history of effective or ineffective treatment with the placebo analgesic cream, which was our main research interest. The placebo conditioning paradigm is widely used to study the effects of expectation on pain by inducing placebo analgesia^17^. Unbeknownst to the subjects, the inert treatment was surreptitiously paired with decreased temperature of the noxious thermal stimulation. It has been shown that placebo effects are induced much more effectively by learning procedures than by falsely informing subjects about the efficiency of treatment because placebo effects depend on learning effects^18^. The placebo was used as the first pain treatment instead of an active pharmacological drug to prevent the initially administered painkiller from affecting the analgesic effect of the following experimental treatment (i.e., cold-pack application). That allowed us to investigate how the efficacy of a subsequent treatment was affected by expectations induced by treatment history without having to control for continued effect of an active drug.

Functional magnetic resonance imaging (fMRI) was used in order to investigate the brain mechanisms underlying the effect of treatment history (manipulated using placebo response conditioning) on the efficacy of cold-pack treatment. This study focused on the cold-pack treatment condition, exploring the difference in behavioral response and neural activity between the negative and positive experience groups. We assumed that the treatment history induced by manipulating the temperature during placebo conditioning would alter patient desire and expectation for the new treatment. Considering previous findings of the positively increased expectation for new treatment after treatment failure^11,14^ and distinguishable features of two treatments which prevent generalization across treatments^13^, we hypothesized that the analgesic effects of cold-pack application would be higher in the negative experience group than in the positive experience group. Such effects may lead to differences in brain activity^1,6,19^ such as deactivation of the brain regions involved in pain perception and activation of the frontoparietal network related to the expectation of pain relief.

## Methods

### Participants

Thirty healthy male volunteers (mean age = 26.40 years, range = 20–39 years) participated and were randomly divided into two groups. Volunteers were recruited through advertisements posted on social networking sites. The inclusion criteria were as follows: right-handed men, 19–39 years of age, without a history of any neurologic or psychiatric disorders. The exclusion criteria were as follows: contraindications to MRI scanning (such as, metallic implants, claustrophobia, or pacemakers), hormonal medication within the past 6 months, use of central nervous system-active medications or illicit drugs, or regular consumption of nicotine or alcohol. The experiment was performed according to the guidelines of the Declaration of Helsinki and approved by the Medical Ethics Committee of Yonsei University, Wonju College of Medicine (CR313017). The informed consent was obtained from participants before the fMRI scanning. The volunteers were paid about 180 US dollars (KRW 200,000) for their participation.

This study used a between-subject design, with subjects assigned to either the positive experience group or the negative experience group; these groups were distinguished by a history of effective or ineffective treatment with the placebo analgesic cream. In our previous study^20^, there was a group difference between the strong placebo and control conditions to be 16.16 on a scale of pain score. The sample size required to have 80% power (i.e., β = 0.2) at α = 0.05 (SD = 20) can be obtained by normal approximation as n = [(Zα/2 + Zβ) × σ/µD]^2^ ≈ 12^21^. To control for a 10% drop rate, the required sample size was 14, resulting in a sample size of 15 in each group. One participant in the negative placebo group was excluded due to excessive head movement during the fMRI scanning.

### Apparatus and materials

**Figure 1:**
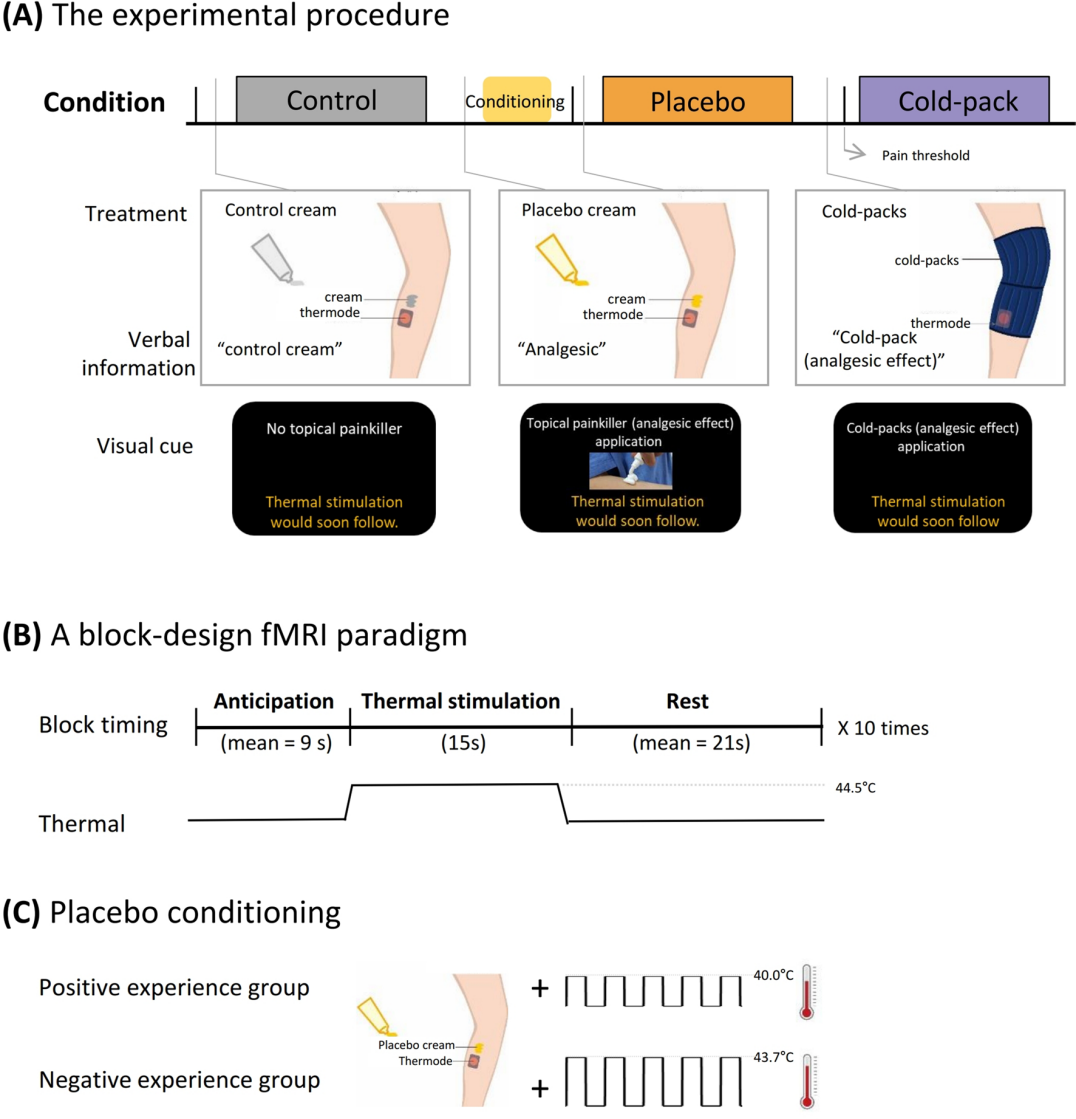
Study protocol. (**A,B**) Noxious thermal stimulation (44.5 °C, 15 s, 5 times) was applied to participants in practice. Following those practice phase, noxious thermal stimulation (44.5 °C, 15 s) in the control condition was repeated ten times during fMRI scanning. (**C**) The thermode temperature in the conditioning session of the placebo condition was surreptitiously reduced from 44.5 to 43.7 °C for weak placebo conditioning (negative experience group) and to 40.0 °C for strong placebo conditioning (positive experience group). Following the weak and strong placebo conditioning sessions, participants in the negative and positive experience groups again received noxious stimulation in the placebo condition during fMRI scanning. After the pain perception experiment in the control and placebo conditions, participants received noxious thermal stimulation in the cold-pack condition during fMRI scanning. Therefore, participants in the positive and negative experience groups received noxious stimulation under three conditions: (1) control, (2) placebo, and (3) cold-pack. The total intensity and duration of noxious stimuli during fMRI scanning were identical in each of the three conditions.

Noxious thermal stimuli were delivered through a computerized thermal contact stimulator (CHEPS, Medoc Advanced Medical Systems Ltd., Ramat Yishai, Israel) with a 27-mm-diameter thermode. To induce the noxious thermal stimuli, the thermode was attached to the participants’ skin above the medial muscle of the left lower leg using a Velcro strap (Fig. 1A). In a previous study, temperatures less than 46 °C (44.5, 45, 45.5, and 46 °C) were used for low noxious thermal stimulation^22^. Normal pain thresholds for a hot stimulus are 44–47 °C^15,23^. Therefore, a temperature of 44.5 °C was set as the noxious stimulation across conditions. In the cold-pack condition, the thermode was attached under the applied gel cold packs (56 cm × 25.5 cm, gel type, DR-IH009, Stanch Ltd., Taipei, Taiwan).

The control (basal) cream applied to the participants in the control condition was ultrasound transmission gel (Care Sonic, Care Pharm Ltd., Ansan, South Korea). For the placebo cream, a small amount of thyme extract (leaf & flower Thyme, Herb Pharm, Williams, OR, USA) was added to the ultrasound transmission gel to produce a brownish color and medicinal smell. The cream that all participants received had no active ingredients. Subjects were not aware that the goal of the experiment was to investigate the effects of placebo analgesia.

The gel cold packs were stored in the freezer compartment of a domestic refrigerator (R-B14FDG 2002, LG Electronics Inc., Yeouido-dong, Seoul, South Korea) until the surface temperature of the thin cloth was maintained at 9 °C, moving it from the freezer compartment to the refrigerator compartment as needed to maintain a temperature of 9 °C. To protect participants’ skin from the low temperature, gel cold packs were put in a sack made of thin cloth (Cham Alzza Sack, Cham Brain Health Institute & Alzza Health Institute, Seoul, South Korea). As shown in Fig. 1A, the left distal thigh and proximal lower leg were wrapped in four cold packs during fMRI scanning.

### Experimental procedure

The experimental procedure is illustrated in Fig. 1A. A practice phase with no fMRI scanning was performed to familiarize the participants with the noxious stimulation procedure. During the practice, a noxious thermal stimulation (44.5 °C, duration = 15 s, five times) was applied. The control cream was applied to the skin of the medial aspect of the left lower leg, but not where the thermode was attached. Each participant underwent the fMRI scanning phase for three experimental conditions: control, placebo, and cold-pack. The total intensity and duration of the noxious stimulus during fMRI scanning were identical (44.5 °C, duration = 15 s, 10 times) in each of the three conditions.

The experiments in the cold-pack condition were always conducted last. The order of the control and placebo conditions was counterbalanced by randomization with constraints, such that half the participants (n = 7 for the negative experience group and n = 8 for the positive experience group) underwent the control condition followed by the placebo condition, and the other half (n = 8 for the negative experience group and n = 7 for the positive experience group) underwent the placebo condition followed by the control condition.

During the placebo conditioning manipulation, the negative and positive experience groups received thermal stimulation five times for 15 s. Unbeknownst to the subjects, the placebo cream was coupled with a low thermode temperature (40.0 °C) delivered to the positive experience group and to a moderate thermode temperature (43.7 °C) in the negative experience group (Fig. 1C). Presuming that temperature manipulations caused the placebo-induced analgesia to vary, the effect of treatment experience history (negative or positive) was compared in the cold-pack condition.

Upon termination of the placebo conditioning procedure, subjects were treated with the placebo cream again and the fMRI scanning phase for the placebo condition was conducted. The participants were informed that the cream was a highly effective pain-relieving medication and short-acting analgesic that would reach its peak effect in around 5 min and then decreased within the next 5 min. In fMRI scanning for the control condition, noxious thermal stimulation was administered while the participant was treated with the control (base) cream, which they were told had no pain relief effect. Lastly, participants underwent the fMRI scanning for the cold-pack condition with the cold-pack treated.

### Noxious stimulation paradigm

We used a block-design fMRI paradigm of anticipation, noxious stimulation, and rest epochs, as shown in Fig. 1B. During the anticipation period, visual instructions about the treatment type (no analgesic cream, applying analgesic cream, or applying a cold pack) were presented as the cue that the noxious stimulation was about to begin. The length of anticipation epochs varied from 6 to 12 s (mean duration = 9 s). The anticipation epoch was followed by the noxious stimulation periodfor 15 s. To allow the blood oxygen level-dependent (BOLD) response to return to baseline, each noxious stimulation was followed by a post-stimulation resting period that varied from 18 to 24 s (mean duration = 21 s). This anticipation-pain-rest cycle was repeated 10 times during the fMRI scanning of each subject in three conditions (control, placebo, and cold-pack), resulting in 10 blocks per condition per subject. The temperature of the noxious stimulus applied to the participants during fMRI scanning was the same in all three experimental conditions.

The visual cues were projected onto a screen in the MRI console that participants could see in a mirror mounted on the head-coil. During the anticipation and noxious stimulation periods in the placebo conditions, the visual cue was Korean text indicating that a topical painkiller (ointment) would be applied to the skin and images showing the direct application of the ointment to the skin. The visual cue used in the control condition was Korean text indicating that no topical painkiller (ointment) would be applied to the skin. During the anticipation and noxious stimulation periods in the cold-pack condition, a visual cue was presented to indicate that the cold-pack had an analgesic effect. However, no comments were provided to the participants comparing the strength of the analgesic effect of analgesic cream (placebo) with that of the cold-pack. Therefore, the expectation that the participants had about the analgesic effect of the cold-pack was examined in this experiment. To minimize the possibility of habituation or sensitization, the thermode was moved a short distance to the adjacent medial sides of each participant’s left lower leg for each of the three conditions.

### Self-reported psychological data

Before the fMRI scan in all three conditions, participants’ thermal pain threshold was tested to measure individual pain sensitivity (Fig. 1A) and to confirm that the treatment history effect between groups was not due to pain threshold difference by condition. Pain threshold describes the lowest temperature at which participants perceived a given noxious stimulus to be painful^24^. The temperature was increased from 32 °C at a rate of 1 °C/s to each participant’s pain threshold level. Because pain perception intensity in a previous study^25^ was significantly reduced with respect to conditioning stimuli at interstimulus intervals below 60 s, 70 s was set as the interstimulus interval in order to determine pain threshold. The final pain threshold was the mean of three pain thresholds obtained during the three consecutive stimulations. Pain threshold in the control condition was tested with no treatment applied to the participant. Pain threshold in the cold-pack condition was tested with the underside of the thermode in contact with the skin surface of the leg, while the top side of the thermode is covered by a cold pack. For the placebo condition, pain threshold was measured right after the placebo cream application. We did not test the difference by condition due to the various environmental measurements for pain threshold, instead focusing on the group difference at each condition.

Upon completion of fMRI scans, participants were asked to rate their average pain intensity, unpleasantness, and anxiety in all three conditions on a numerical rating scale (NRS; 0 = no pain, not unpleasant, and no anxiety; 100 = maximum imaginable pain, highly unpleasant, and severe anxiety).

### Statistical analyses of behavioral data

Behavioral data were analyzed using SPSS software. Differences between the placebo intervention groups in self-reported behavioral data of the placebo analgesic effect [control—placebo condition] and the cold-pack condition were tested using two-sample t-testing.

In addition, we performed a one-way repeated measures analysis of variance (RM-ANOVA) to investigate the condition effects among the control, placebo, and cold-pack conditions. When a significant difference or condition effect was observed, Bonferroni post hoc tests were performed. If the score did not meet the established assumption of normality (Kolmogorov–Smirnov test), Mann–Whitney U and Friedman testing were used for further analysis. For all tests, the probability level for statistical significance was set at α = 0.05.

### Functional imaging and analysis

#### MRI acquisition

Before scanning, participants were instructed to stay awake and refrain from moving throughout the imaging session. After participants were in a comfortable position, their heads were immobilized with padded earmuffs and a foam headrest, and a plastic bar was placed across the bridge of the nose. MRI data were acquired using a 3 Tesla (3 T) MRI scanner (Philips Medical Systems, Best, Netherlands). Functional images were acquired using echo-planar imaging with the following imaging parameters: echo time = 35 ms, repetition time = 3000 ms, flip angle = 90°, matrix size = 128 × 128, field of view = 220 × 220 mm^2^, voxel size = 1.72 × 1.72 × 4 mm^3^, gap = 0.5 mm, and slice thickness = 4 mm. Thirty-three slices were acquired to include the entire brain volume. A structural T1-weighted image was obtained using a gradient echo sequence (echo time = 4.6 ms, repetition time = 9.9 ms, flip angle = 8°, matrix size = 220 × 220, field of view = 220 × 220 mm^2^, and voxel size = 1 mm^3^).

#### Image preprocessing

Preprocessing and statistical analysis of task-based fMRI data were conducted using Statistical Parametric Mapping (SPM12, http://www.fil.ion.ucl.ac.uk/spm) implemented in MATLAB (MathWorks, Inc., Concord, MA, USA). First, functional images were realigned to the mean image for correcting head movements, resulting in head motion-corrected functional images as well as the mean functional image. Head motion was restricted to < 1.5 mm of displacement or 1 degree of rotation in any direction. One participant was excluded from further analysis due to excessive head motion. Second, the individual structural image was then registered to the mean functional image, and the resulting structural image was subjected to segmentation. Functional images were normalized to the Montreal Neurological Institute (MNI) 152-brain template using the transformation matrix obtained in a previous step. Images were resampled into isotropic 2 mm^3^. Finally, normalized fMRI images were spatially smoothed with 6-mm full-width half-maximum. The outputs of six head-motion parameters were used for the first-level individual analysis, as well as for the exclusion of one participant.

#### Statistical analysis

At the first level, preprocessed fMRI data were evaluated using a general linear model and a block fMRI design in which each regressor was modeled as a boxcar function convolved with the canonical hemodynamic response function. We defined six regressors, an anticipation period and a thermal pain stimulation period for each of the three conditions. The six head-motion parameters, three translations and three rotations, derived from the realignment procedure, were entered as covariates of no interest to remove the effects of head motion. To remove low-frequency drift and control for serial correlations, temporal high-pass filtering with a cut-off frequency of 1/128 Hz and a first-order autoregressive model (1) were applied to the preprocessed fMRI data. Contrast images of the six regressors were produced and subjected to second-level random-effects group analysis.

Two-sample t-testing was conducted on the cold-pack contrast images to test the effects of the placebo intervention experience on brain activation during the pain anticipation and pain delivery periods under the cold-pack condition. Statistical parametric maps were primarily thresholded at a voxel-level p value of 0.001. To correct for multiple comparisons, a cluster-extent threshold calculated by the Gaussian random field method was implemented in SPM12, resulting in a cluster-level family-wise error-corrected *p* value of 0.05. The resulting statistical map was superimposed on the MNI template provided by MRIcron software (http://www.nitrc.org/projects/mricron). The clusters demonstrating significant group difference was labeled in accordance with the SPM Anatomy toolbox v2.1^26^. The % BOLD signal change from the significant cluster during the cold-pack condition was extracted using Marsbar (http://marsbar.sourceforge.net/), then plotted each group for visualization. We also extracted the percent signal change value under the placebo condition from significant clusters to explore group differences in the effect of treatment history on the cold-pack condition, to determine whether this is also attributable to the placebo condition.

## Results

### Demographics and self-reports

The demographic and self-report scale score for the cold-pack condition are presented in Table 1. The negative and positive experience groups did not differ in age, weight, or height. Behavioral placebo analgesic effects [control—placebo condition] on the self-reported pain threshold (°C), pain intensity, anxiety, and unpleasantness ratings are depicted in Supplementary Fig. S1. Placebo analgesic effects and placebo-induced unpleasantness reductions were significantly stronger in the positive experience group than in the negative experience group.

**Table 1:** Demographic and self-reports characteristics of the cold-pack condition in the negative and positive experience group.

Variables	Negative experience group	Positive experience group	statistics
**Demographic variables**			
Age, years	27.07 ± 7.949	26.2 ± 5.4	*t* = 0.34, *p* = 0.735
Weight	72.5 ± 14.5	73.9 ± 8.9	*t* = − 0.31, *p* = 0.760
Height	173.6 ± 5.7	177.7 ± 5.7	*t *= − 1.99, *p* = 0.057
**Cold-pack condition (self-reported data)**			
Pain threshold	47.29 ± 0.99	46.93 ± 0.88	*t* = − 1.01, *p* = 0.321
Pain rating	47.14 ± 16.02	61.33 ± 18.17	*t* = − 2.22, *p* = 0.035
Anxiety	33.21 ± 19.07	47.67 ± 18.21	*t *= − 2.09, *p* = 0.047
Unpleasantness	35.36 ± 18.76	50.00 ± 20.70	*t* = − 1.99, *p* = 0.057

Values are presented as the mean ± standard deviation.

#### Difference between the negative and positive experience groups in the cold-pack conditions

**Figure 2:**
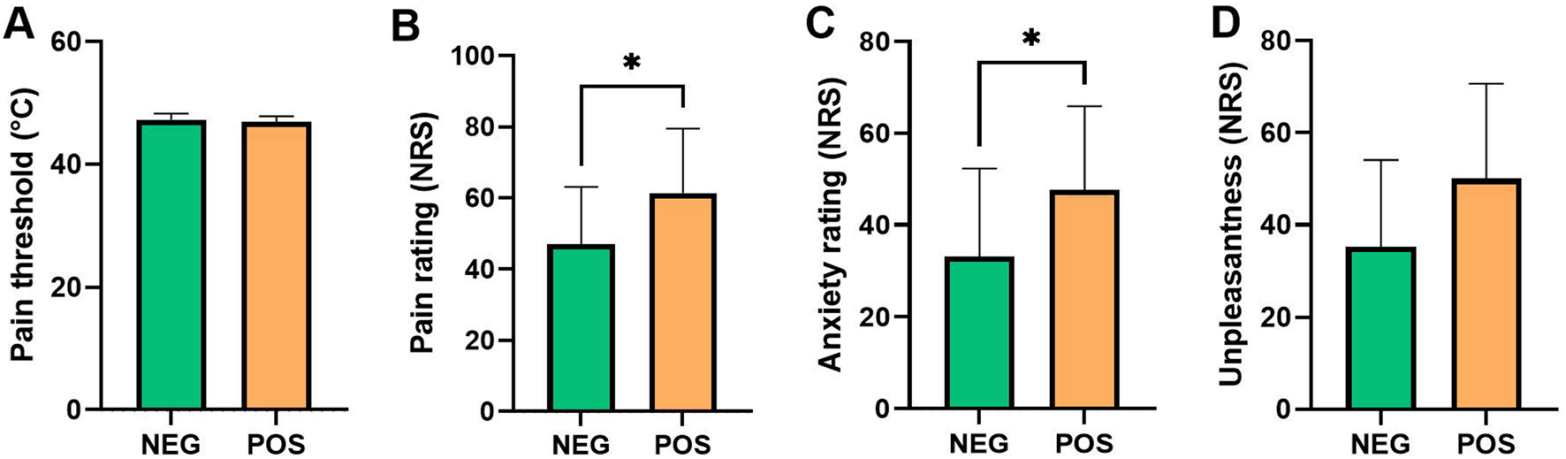
Changes in pain threshold (°C), pain, anxiety, and unpleasantness ratings in the cold-pack condition. (**A**) The pain threshold (°C) did not differ between the negative and positive experience groups. (**B,C**) Pain and anxiety scores were lower in the negative experience group than in the positive experience group. (**D**) Unpleasantness did not differ between the negative and positive experience groups. *NEG* negative experience group, *POS* positive experience group, *NRS* numeric rating scale.

In the cold-pack condition, the negative experience group reported a significantly lower pain rating than the positive experience group, *t* =  − 2.22, *p* = 0.035 (Fig. 2B). The reported anxiety score of the negative experience group was also significantly lower than that of the positive experience group, *t* =  − 2.087, *p* = 0.047 (Fig. 2C). A marginal group difference was found in the unpleasantness rating, with the unpleasantness reported by the positive experience group being slightly higher than that reported by the negative experience group, *t* =  − 1.99, *p* = 0.057 (Fig. 2D). No significant group difference was found in pain threshold in the cold-pack condition (Fig. 2A).

#### Differences among the three conditions

Next, differences in self-reported measurements among the three conditions were analyzed. For anxiety scores, statistically significant differences among conditions were observed, *F*(2,56) = 8.077, *p* < 0.001. Anxiety scores during the anticipation periods were significantly higher in the control condition (mean ± SD = 52.9 ± 16.3) than in the placebo (41.0 ± 18.4, *p* = 0.0093) and cold-pack (40.7 ± 19.7, *p* = 0.001) conditions.

Subjective pain ratings also differed significantly among the three conditions,*F*(2,56) = 6.962, *p* = 0.002. Post-hoc tests using the Bonferroni correction revealed that pain ratings were significantly higher in the control condition (60.2 ± 12.5) than in the placebo condition (47.4 ± 15.9) during the noxious stimulation periods (*p* = 0.0006). Pain ratings did not differ significantly between the cold-pack condition (54.48 ± 18.34) and the control or placebo conditions during the noxious stimulation periods.

Unpleasantness ratings also showed statistically significant condition differences, *F*(2,56) = 8.77, *p* = 0.0005. Post-hoc tests demonstrated that unpleasantness ratings in the control condition (55.34 ± 14.20) were significantly higher than in the placebo condition (40.34 ± 18.02, p = 0.0005) and the cold-pack condition (42.93 ± 20.81, *p* = 0.0053) during the noxious stimulation periods.

### Imaging results

**Figure 3:**
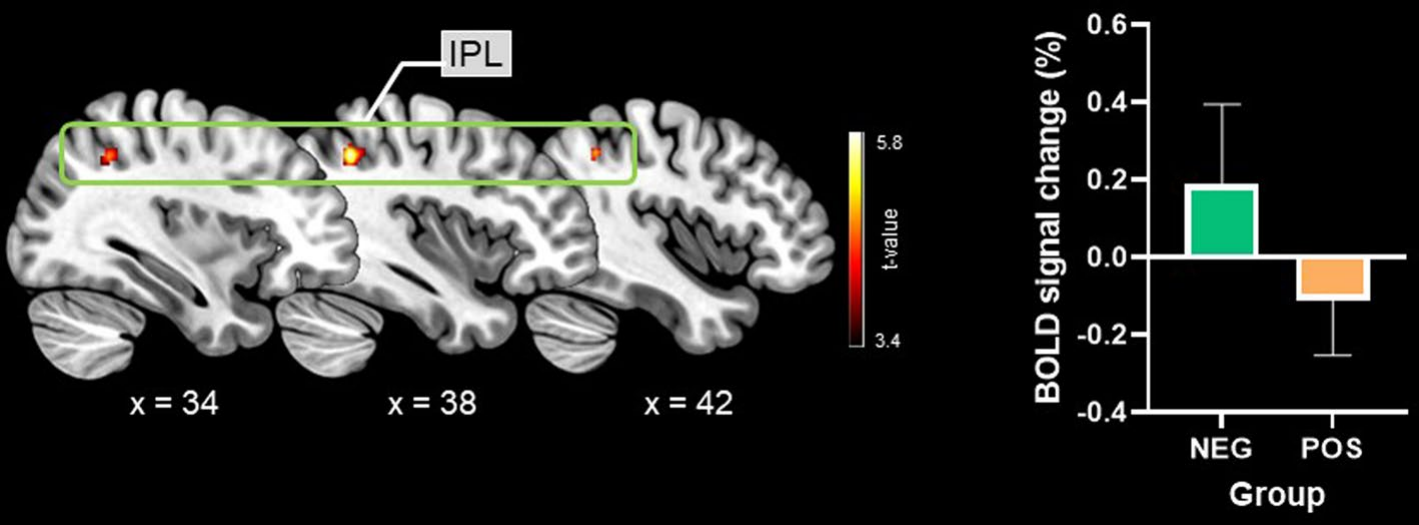
Group differences in fMRI findings in the cold-pack condition. Significant group differences in the cold pack condition were found in the inferior parietal lobule, showing greater activation in the negative experience group and deactivation in the positive experience group. *NEG* negative experience group, *POS* positive experience group, *IPL* inferior parietal lobule.

The placebo conditioning exposure effects on the cold-pack condition were examined by performing a two-sample t-test between the negative and positive experience groups during pain processing in the cold-pack condition. A significant group difference for the cold-pack condition was found in the right IPL (MNI coordinates x = 38, y =  − 52, z = 42, k = 194). As shown in Fig. 3, the right IPL showed greater activation in the negative experience group and deactivation in the positive experience group. Further ROI-based analysis revealed that the % BOLD signal change of the right IPL region during the placebo condition did not significantly differ between groups (mean ± SD, NEG: 0.065 ± 0.173; POS: − 0.058 ± 0.230, *p* = 0.118). During the anticipation periods of the cold-pack condition, no significant group differences were found.

## Discussion

In the current study, we investigated the effect of treatment history on the efficacy of subsequent cold-pack treatment. Treatment failure with placebo cream increased the pain reduction of the following cold-pack treatment, indicating the increased efficacy of a different treatment after previous history of failure. Moreover, negative experience group reported lower emotional aspect of pain such as anxiety and unplesanteness (marginally) during the cold-pack condition than the positive experience group. Our neuroimaging findings of the cold-pack condition demonstrated that the significantly increased brain activation in the negative experience group compared with the positive experience group was found in right IPL.

In the current study, prior treatment with placebo cream between groups was manipulated using placebo conditioning procedures. We replicated the placebo analgesic effect induced by placebo conditioning, with the positive experience group reporting greater reduction in pain rating and unpleasantness score compared with the negative experience group in the placebo condition versus the control condition. However, we did not directly measure participant expectation of the placebo cream, which limits our ability to differentiate the effects of conditioning and expectation of this placebo analgesic effect. Our finding of a behavioral placebo effect [control—placebo condition] showed that the two group had different experiences with the originally applied pain relief treatment.

Increased analgesic effect of the cold-pack treatment after experiencing the treatment failure was found in this study. The pain and anxiety scores during the periods of noxious stimulation in the cold-pack condition were significantly lower in the negative experience group than in the positive experience group. The pain thresholds did not differ between the groups, suggesting that differing pain ratings between the groups resulted not from any sensory-discriminative component of pain, but from cognitive and affective components of pain. Our results seem to contradict previous studies^9,11^ showing the carry-over effect of previous treatment history across treatments with different drugs and treatment characteristics. Recent studies have also shown the generalization effect on the placebo analgesic response^12,13^, with gradients of a similarity-based generalization. Unlike previous studies used similar treatments, we used pharmacological cream and non-pharmacological treatment. Cold-pack treatment did not share any common feature with the placebo cream. These properties might prevent the carry-over effect of treatment history from cream to cold-pack. Although we did not measure participant expectation of the cold pack, previous studies have shown that the expectation of a new treatment positively increases after experiencing treatment failure^11,14^. Similarly, this positive expectation might increase for the participant who underwent unsuccessful treatment experience with the placebo cream and was aware of the analgesic effect of the subsequent treatment with a cold pack. The analgesic effect of a cold pack is mild to moderate^27,28^ and is widely used for pain relief in a broad range of medical areas. Considering these previous findings, participants with unsuccessful treatment experience of the placebo cream may elicit greater expectation of pain relief from new treatment than the participant with successful previous treatment history, which in turn increase the therapeutic efficacy of the cold-pack treatment.

Our neuroimaging data showed significant activation differences as a function of treatment history in the IPL region, which is involved in constructing top-down representations of context, such as beliefs and expectations regarding placebo analgesia. A recent study reported that the intraparietal sulcus, which includes the right IPL, displayed increased pain-related activity when pain relief was expected^7^. In the current study, the group that experienced an unsatisfying previous treatment exhibited greater right IPL activation during pain processing with the new treatment (i.e., cold pack) along with the greater analgesic response than the group that experienced a satisfying previous treatment. In addition, the increased IPL activity associated with analgesic effect was not observed during the placebo condition. This finding indicates that treatment expectations evoked by previous treatment experience could differ depending on the previous exposure of the treatment.

Evidence suggests that the lateral intraparietal area (LIP), a subdivision of the IPL, is involved in decision making^29,30^. Reward‐related decision-making relies on motivation, evaluation of the rewards and punishments associated with different options, and responsiveness to the available rewards^31^. When the reward size is varied in animal models, LIP neurons’ activity is more prominent when larger rewards are presented and smaller when smaller rewards are presented^32^. The activity of LIP neurons is thus interpreted to reflect the amount of potential reward. To obtain the highest expected value (reward), decision-making is based on the product of the expected reward and its probability^32^. In the present study, subjects in the negative experience group did not experience pain relief (reward) from the first placebo administered, so they expected pain relief (reward) from the cold-pack treatment that they received second. The expectation of that pain relief might have led to an increase in the activity of the right IPL in those subjects under the cold-pack condition.

Although most neuroimaging studies to date have focused on BOLD activations in response to noxious stimuli, several studies have reported pain-induced fMRI or PET (Positron Emission Tomography) deactivations in several brain regions, including the IPL, ventral medial prefrontal cortex, and posterior cingulate, which are all core DMN structures^33,34^. Pain-induced fMRI or PET deactivation in response to noxious stimuli cannot be definitely explained, but it probably provides important information. The DMN is active during the rest or baseline state, whereas it is suppressed during various attention-demanding (cognitive-perceptual) tasks^35^. Therefore, various attention-demanding tasks cause task‐independent deactivation of the DMN, compared to the baseline state^35^. In the cold-pack condition, the positive experience group reported a higher pain score than the negative experience group, so participants in the positive experience group would have been more likely to attend to their pain, causing IPL deactivation.

In addition, a previous study using 7 levels of pain intensity found that the activity of the right IPL and posterior-lateral temporal cortex activity decreased as the pain intensity increased^36^. In that study, Loggia et al. applied 14 s of pressure to the left calf, and the right (contralateral to the noxious stimulus) IPL and posterior-lateral temporal cortex were linearly deactivated as pain increased. Loggia et al.’s study and our present study have several similarities, such as the part of the body (left calf) to which the noxious stimulation was applied, the duration of the noxious stimulation, and the deactivation of the right (contralateral to the pain stimulus) IPL as pain increased. On the 0–100 numerical pain intensity scale (0 = no pain, 100 = the most intense pain tolerable), pain intensity ratings of 10, 20, 30, and 40 showed positive % BOLD signal changes, whereas pain intensity ratings of 50, 60, and 70 showed negative % BOLD signal changes in Loggia et al.’s study^36^. In the cold-pack condition of the present study, pain intensity ratings in the negative experience group were 47.1 ± 16.0, whereas those in the positive experience group were 61.3 ± 18.2. In this study, as in Loggia et al.’s study, negative % BOLD signal changes were shown at a mean pain intensity rating of 61.3, whereas positive % BOLD signal changes were shown at a mean pain intensity rating of 47.1. The fact that Loggia et al.’s research findings and our research findings are similar demonstrates the reliability of our research and indicates that our research findings can be generalized.

The first limitation of this study was that the study included only male participants because of the higher sensitivity to pain in women than men^37,38^. Considering sex differences in placebo analgesia, there may be limitations in generalizing the findings of this study to women. The second limitation of this study is that this study was not conducted on pain patients but on healthy volunteers, so there may be limitations in applying the findings of this experiment to actual clinical patients. However, human experimental pain models in healthy volunteers can act as a translational bridge between animal and clinical research^39^. Therefore, the findings of this experiment in healthy volunteers may still be relevant to clinical pain patients.

In conclusion, our present results suggest that people treated with painkillers that have potent analgesic effects could experience weak analgesic effects from their next pain treatment. Conversely, people treated with painkillers that have mild analgesic effects could experience potent analgesic effects from their subsequent pain treatment. In our behavioral findings, the pain ratings in the cold-pack condition were significantly lower in the negative experience group than in the positive experience group, and those results were supported by our imaging results, which demonstrated that the right IPL was significantly more active in the negative experience group than in the positive experience group. Our experiment thus suggests that when the analgesic effect of currently used painkillers is not significant, human brains (specifically, areas in the IPL) attribute more potent analgesic effects to subsequent treatments. This experiment also suggests that when pain is not relieved by certain medications, the analgesic effects of following pain treatments could be enhanced by activation of the IPL.

## Supplementary Information

Supplementary Figure S1.

## Data Availability

The datasets used and/or analysed during the current study are available from the first author and corresponding author upon reasonable request.

## References

[1] Colloca L, Klinger R, Flor H, Bingel U (2013). Placebo analgesia: Psychological and neurobiological mechanisms. Pain.

[2] Atlas LY, Wager TD (2012). How expectations shape pain. Neurosci. Lett..

[3] Bingel U (2011). The effect of treatment expectation on drug efficacy: Imaging the analgesic benefit of the opioid remifentanil. Sci. Transl. Med..

[4] Amanzio M, Benedetti F (1999). Neuropharmacological dissection of placebo analgesia: Expectation-activated opioid systems versus conditioning-activated specific subsystems. J. Neurosci..

[5] Scott DJ (2008). Placebo and nocebo effects are defined by opposite opioid and dopaminergic responses. Arch. Gen. Psychiatry.

[6] Petrovic P, Kalso E, Petersson KM, Ingvar M (2002). Placebo and opioid analgesia—Imaging a shared neuronal network. Science.

[7] Zunhammer M, Spisak T, Wager TD, Bingel U, Placebo Imaging C (2021). Meta-analysis of neural systems underlying placebo analgesia from individual participant fMRI data. Nat. Commun..

[8] Reicherts P, Gerdes AB, Pauli P, Wieser MJ (2016). Psychological placebo and nocebo effects on pain rely on expectation and previous experience. J. Pain.

[9] Kessner S (2014). The effect of treatment history on therapeutic outcome: Psychological and neurobiological underpinnings. PLoS ONE.

[10] Kessner S, Wiech K, Forkmann K, Ploner M, Bingel U (2013). The effect of treatment history on therapeutic outcome: An experimental approach. JAMA Intern. Med..

[11] Zunhammer M (2017). The effects of treatment failure generalize across different routes of drug administration. Sci. Transl. Med..

[12] Kampermann L, Tinnermann A, Büchel C (2021). Generalization of placebo pain relief. Pain.

[13] Liu C, Chen L, Yu R (2019). Category-based generalization of placebo and nocebo effects. Acta Psychol. Amst..

[14] Müller M (2016). Treatment history and placebo responses to experimental and clinical pain in chronic pain patients. Eur. J. Pain.

[15] Saeki Y (2002). Effect of local application of cold or heat for relief of pricking pain. Nurs. Health Sci..

[16] Martin-Pichora AL, Mankovsky-Arnold TD, Katz J (2011). Implicit versus explicit associative learning and experimentally induced placebo hypoalgesia. J. Pain Res..

[17] Price DD (1999). An analysis of factors that contribute to the magnitude of placebo analgesia in an experimental paradigm. Pain.

[18] Petrovic P (2005). Placebo in emotional processing-induced expectations of anxiety relief activate a generalized modulatory network. Neuron.

[19] Zunhammer M, Bingel U, Wager TD (2018). Placebo effects on the neurologic pain signature: A meta-analysis of individual participant functional magnetic resonance imaging data. JAMA Neurol..

[20] Choi JC (2017). Testosterone effects on pain and brain activation patterns. Acta Anaesthesiol. Scand..

[21] Shein-Chung C, Jun S, Hansheng W (2007). Sample Size Calculations in Clinical Research, 2 edn.

[22] Hashmi JA, Davis KD (2010). Effects of temperature on heat pain adaptation and habituation in men and women. Pain.

[23] Verdugo R, Ochoa JL (1992). Quantitative somatosensory thermotest. A key method for functional evaluation of small calibre afferent channels. Brain.

[24] Choi JC (2013). Changes in pain perception and hormones pre- and post-kumdo competition. Horm. Behav..

[25] Schestatsky P (2007). Transient decrease of sensory perception after thermoalgesic stimuli for quantitative sensory testing. Muscle Nerve.

[26] Eickhoff SB (2005). A new SPM toolbox for combining probabilistic cytoarchitectonic maps and functional imaging data. Neuroimage.

[27] Exton DR, Fenner PJ, Williamson JA (1989). Cold packs: Effective topical analgesia in the treatment of painful stings by Physalia and other jellyfish (for editorial comment, see page 610; see also pages 621, 626 and 708). Med. J. Aust..

[28] Malanga GA, Yan N, Stark J (2015). Mechanisms and efficacy of heat and cold therapies for musculoskeletal injury. Postgrad. Med..

[29] Huk AC, Katz LN, Yates JL (2017). The role of the lateral intraparietal area in (the study of) decision making. Annu. Rev. Neurosci..

[30] Sugrue LP, Corrado GS, Newsome WT (2005). Choosing the greater of two goods: Neural currencies for valuation and decision making. Nat. Rev. Neurosci..

[31] McGovern AR, Alexopoulos GS, Yuen GS, Morimoto SS, Gunning-Dixon FM (2014). Reward-related decision making in older adults: Relationship to clinical presentation of depression. Int. J. Geriatr. Psychiatry.

[32] Platt ML, Glimcher PW (1999). Neural correlates of decision variables in parietal cortex. Nature.

[33] Buckner RL, Andrews-Hanna JR, Schacter DL (2008). The brain's default network: Anatomy, function, and relevance to disease. Ann. N. Y. Acad. Sci..

[34] Kong J (2010). Exploring the brain in pain: Activations, deactivations and their relation. Pain.

[35] van Oudenhove L (2009). Cortical deactivations during gastric fundus distension in health: Visceral pain-specific response or attenuation of 'default mode' brain function? A H2 15O-PET study. Neurogastroenterol. Motil..

[36] Loggia ML (2012). Disentangling linear and nonlinear brain responses to evoked deep tissue pain. Pain.

[37] Casale R (2021). Pain in women: A perspective review on a relevant clinical issue that deserves prioritization. Pain Therapy.

[38] Choi JC (2016). Brain mechanisms of pain relief by transcutaneous electrical nerve stimulation: A functional magnetic resonance imaging study. Eur. J. Pain.

[39] Kumar Reddy KS, Naidu M, Rani PU, Rao TRK (2012). Human experimental pain models: A review of standardized methods in drug development. J. Res. Med. Sci..

