# Supplementary material for: The increased analgesic efficacy of cold therapy after an unsuccessful analgesic experience is associated with inferior parietal lobule activation
Source: Sci Rep. 2022 Aug 29;12:14687. doi: 10.1038/s41598-022-18181-0 (PMC9424269; doi:10.1038/s41598-022-18181-0)
Supplement: Supplementary file 1 — Supplementary Figure S1. [file 41598_2022_18181_MOESM1_ESM.docx]

Supplementary Information


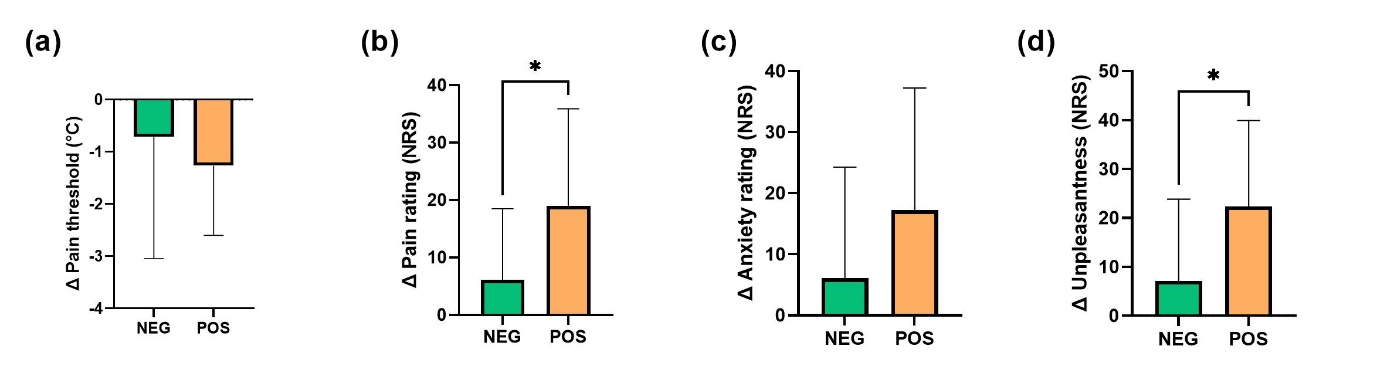


**Supplementary Figure S1.** **Behavioral placebo effects [control – placebo condition] in the self-reported pain threshold (°C), pain intensity, anxiety, and unpleasantness ratings. NEG** = negative experience group; POS = positive experience group; NRS = numerical rating scale.

(a) Δ Pain threshold was expressed as a degree difference by subtracting the pain threshold for the placebo condition from the pain threshold for the control conditions. Behavioral placebo effects reported on a 0-100 NRS for (b) pain intensity, (c) anxiety, and (d) unpleasantness were calculated by subtracting the response in the placebo condition from the response in the control condition. A significant group difference was found in pain intensity and unpleasantness rating, with the positive experience group reporting greater analgesic effects of the placebo cream and less placebo-induced unpleasantness compared with the negative experience group.
